# Feasibility and Efficacy of a Psychological Therapy for Patients With a Schizophrenic Psychosis in an Inpatient Setting: Study Protocol of a Randomized Switch Controlled Trial

**DOI:** 10.3389/fpubh.2020.00391

**Published:** 2020-08-12

**Authors:** Mona Redlich Bossy, Daniel Mueller, Erich Seifritz, Stefan Vetter, Stephan T. Egger

**Affiliations:** ^1^Department of Psychiatry, Psychotherapy and Psychosomatics, Faculty of Medicine, Psychiatric University Hospital of Zurich, University of Zurich, Zurich, Switzerland; ^2^Department of Psychiatry, Faculty of Medicine, University of Bern, Bern, Switzerland

**Keywords:** schizophrenic psychosis, randomized controlled trial, inpatient, CBT for groups, NCR, INT, IPT

## Abstract

**Background:** Schizophrenic psychoses are severe mental disorders. Despite advances in treatment, outcomes are still unsatisfactory. Pharmacological treatments are still limited, in particular regarding improvements in psychosocial functioning and neuro-cognitive impairment. In recent years new psychological therapies have been developed, demonstrating promising results. However, most of these interventions have been designed for and studied in outpatients; their efficacy and feasibility for patients requiring hospitalization is still unknown. Therefore, we have designed a clinical trial to compare a neuro-cognitive (Integrated Neuro-cognitive Treatment INT); a cognitive-behavioral (Integrated Psychological Therapy IPT); and a control (Cogpack CGP) intervention for patients with a schizophrenic psychosis hospitalized for treatment.

**Methods:** In a three-parallel-arm, single-blind, randomized, controlled study, we compare INT, IPT, and CGP. Participants will take part in two weekly sessions of one intervention for at least 16 sessions. If due to randomization, participants are allocated to a treatment arm not suitable for them, they are allowed to switch intervention after four sessions. Based on a sample size calculation, recruitment will continue until 30 participants have completed the intervention for each treatment arm.

**Outcome Measurement:** Primary outcomes are: change in symptom as measured by the Positive and Negative Syndrome Scale (PANSS), change in psychosocial functioning as assessed by the mini ICF-APP and neuro-cognitive performance, assessed by the Matrics Cognitive Consensus Battery (MCCB). Other outcomes of interest are the Brief Symptom Inventory (BSI) and the Health of the Nation Outcome Scales (HoNOS); together with prescribed medication, treatment retention and completion rates. Outcomes will be measured at baseline, 2 weeks into treatment (prior to a potential switch of intervention arm), post-treatment and at 6 and 12-month post-treatment follow-ups.

**Expected Outcomes:** We expect an overall improvement; however, with differences in specific domains for each treatment arm, with those completing INT showing better outcomes than IPT and CGP, respectively. We anticipate that lower functioning participants will drift to CGP and higher functioning participants to INT.

**Conclusion:** Due to the complexity of treatment for patients with a schizophrenic psychosis, we consider it crucial to compare different treatment options for those more severely affected, therefore, requiring inpatient treatment.

**Trial registration:** www.clinicaltrials.gov (ID: NCT03316664; 17.10.2017).

## Background

Schizophrenic psychoses are severe mental disorders, with a heterogeneous combination of symptoms and a lifetime prevalence of around one per cent (1, 2). Characteristic symptoms, albeit not exclusive, are hallucinations, delusions, apathy, blunting of affect, disorganized speech and thinking, together with cognitive impairment (3). Those affected are struck in the prime of life and are frequently unable to cope with the challenges of everyday life; experiencing impairment and disability in multiple domains, including the ability to maintain social relationships, sustain employment, and live independently (1, 2).

The treatment of schizophrenic psychoses remains a challenge; only around half of the patients show substantial clinical improvement (4). Treatment of Schizophrenia still relies predominantly on antipsychotic agents (5). Since their clinical introduction over 60 years ago, the Dopamine D2 receptor antagonism remains the pivotal mechanism of action. Newly developed antipsychotics follow the strategy of maintaining this effect, while attempting to improve tolerability (5). The pharmacological treatment of schizophrenia is often limited due to side-effects (6–8). Overall, antipsychotic agents are most effective in reducing positive symptoms, in comparison other symptom domains, such as psychosocial functioning, negative symptoms, and cognition show minimal improvement (5, 9).

In the past few decades, psychological interventions have been shown to be effective when used in conjunction with pharmacological treatment (10, 11). Psychoeducation, assertiveness training, family therapy, cognitive behavioral therapy, and cognitive remediation treatment programs have been developed and systematically studied and further improved (10–15). Current guidelines recognize their importance for treatment and outcome, correspondingly implementation early on in treatment is recommended, even in hospitalized patients (16–19). However, current evidence regarding the effectiveness of psychotherapeutic interventions has been obtained primarily in studies of outpatient populations; studies in chronic and low functioning patients hospitalized for treatment are sparse (20, 21), with patient recruitment a major challenge (22).

The Integrated Psychological Therapy (IPT) for patients with schizophrenia is one of the first manualized integrated cognitive remediation therapy programs for groups, combining social skills, neuro- and social cognition in a single therapy (23, 24). Since the first trial in 1980, IPT has been extensively studied, with empirical evidence consistently demonstrating its efficacy; consequently, it is currently implemented in clinical practice (25). The further development of IPT has led to Integrated Neurocognitive Therapy (INT) for Schizophrenia, a manualized cognitive remediation therapy, which includes a computerized neurocognitive training component (Cogpack CGP) (26, 27). In clinical trials INT has been shown to not only improve neuro- social-cognitive performance but also to have the potential to improve functional outcome and to reduce negative symptoms (24, 27–29).

Although both INT and IPT seem to be effective treatments for patients with schizophrenia; the therapeutic approaches differ in terms of the symptoms they address, together with how the intervention is delivered (29, 30). Through its plain and straightforward design, IPT is suitable for chronic and hospitalized patients (31). However, IPT does not target all the cognitive domains impacted in schizophrenia (32). In contrast, INT not only integrates these cognitive domains in its therapeutic approach, it also includes elements designed to improve self-awareness and perception of the environment. Furthermore, it includes a computer-based cognitive training, with effects of its own. Consequently, INT is a more complex and challenging intervention than IPT for both therapists and participants (29). Primarily for this reason, INT trials to date have been conducted almost exclusively in outpatient settings (29, 30).

Generally, outpatients are less severely ill (33), which may have implications regarding the implementation of INT for patients requiring hospitalization for treatment. To the best of our knowledge, there have been no trials directly comparing IPT to INT; it is therefore not known if both therapy approaches are equally effective or if one program works better for certain patients or is indeed detrimental for others. Important factors which need to be taken into account are the symptom load and severity in patients requiring hospitalization for treatment, the conditions of the treatment unit, in particular fluctuation in the patient population, together with the presence of patients with disturbing behavior (21).

Therefore, we plan to conduct a single-blind, randomized, controlled trial comparing INT, IPT, and CGP in patients with schizophrenia requiring hospitalization for treatment; to assess the efficacy and feasibility of INT as a treatment programme for such patients. The main outcomes for the assessment of efficacy are changes in symptom load and functionality, together with cognitive performance. Outcomes for feasibility are retention, switch rates, and overall therapy attendance.

## Methods

### Design

We have designed an 8-week, randomized, controlled, assessor-blind, three-parallel-arm trial for patients diagnosed with a schizophrenic psychosis. All patients with the diagnosis of a schizophrenic psychosis (according to the DSM 5 Diagnostic criteria) are eligible to participate. In order to allow the participation of chronic and low-functioning patients, the inclusion criteria were deliberately broad (see Table 1).

**Table 1:** Inclusion and Exclusion criteria.

**Inclusion criteria**
Participants are competent to give informed consent, as determined by the referring physician or psychiatrist.
Diagnosis of Schizophrenia or Schizoaffective Disorder according to DSM-5 (34)
Participants are between 18 and 65 years of age.
Completion of regular compulsory education.
German language proficiency as a native speaker or level B1 Common European Framework of Reference for Languages (CEFRL) (35)
**Exclusion criteria**
Unwilling or unable to comply with study instructions.
Low Intelligence as confirmed by failure to complete regular compulsory education.
Currently in another psychotherapeutic treatment, either in individual or group sessions.
Current consumption of alcohol or illicit drugs.

### Recruitment

The Center for Integrative Psychiatry [ZIP: (German) Zentrum für Integrative Psychiatrie], is part of the Psychiatric University Hospital of Zurich specializes in treating “heavy-users,” i.e., those patients with frequent or long-term hospitalizations for whom outpatient treatment is often insufficient. Participants will be recruited among the patients hospitalized for treatment in the Unit for Psychotic Disorders of the ZIP.

### Interventions and Therapists

Cogpack is a computer-based neuropsychological cognitive training program, covering seven domains: visual-motor skills; processing speed; vigilance; executive functions; memory; verbal comprehension and problem- solving (26, 36). Tasks employed will be identical to those used in the INT sessions. The difficulty level of each task is adapted automatically by the program. At the end of each task, the computer program generates a feedback-report for every user on their performance including “percentage correct” and “completion speed.” Cogpack will be delivered by a psychiatric medical resident, with cognitive behavioral training and a 2-h introduction to the computer program followed by practical training.

Integrated Psychological Therapy (IPT) is a manualized psychological intervention consisting of five modules (37). The five modules are hierarchically arranged and deal with cognitive deficits, perceptual deficits, verbal communication, social skills, and problem-solving, with successively increasing complexity in each module. The procedure and contents for each module are broadly predefined and can be adapted to each group's specific needs. Therapists delivering IPT are nurses (as IPT has been traditionally delivered) with basic training in cognitive behavioral methods and a 3-day seminar on IPT followed by practical training and regular supervision.

Integrated Neurocognitive Therapy (INT) is a manualized psychological intervention consisting of four modules. Each module consists of neuro- and social-cognitive elements, together with stress and emotional regulation domains. The complexity of the modules increases successively as do the emotional demands. INT has a strategy-based-learning and a drill-and-practice approach, where the same didactic structure is applied to each domain (27, 38). One peculiarity feature of INT is the integration of cognitive domains, together with self-awareness/perception; and computer exercises (CGP) (26). Therapists delivering INT have a psychology and psychotherapy degree and have attended a 3-day seminar on INT, followed by practical training and receive regular supervision.

Taking into account the usual length of stay in our unit, together with current recommendations, we have adapted both IPT and INT to 20 session programmes comprising the contents of all modules, whilst respecting the specifications and recommendations of the manuals. Cogpack modules are analogous to those of INT. For an overview of the parallel interventions according to TIDieR (see Table 2).

**Table 2:** Content and chedule of Interventions (ToM: Theory of Mind).

	**IPT**	**INT**	**CGP**
**First Block**	**Module 1**	**Module A**	**Module A**
Sesion 1	Card sorting	Information processing	Information processing
Sesion 2	Verbal concept	Attention/vigilance	Attention
Sesion 3	Verbal concept	Perception of emotions	Attention
Sesion 4	Search strategies	Perception of emotions	Vigilance
**Second Block**	**Module 2**	**Module B**	**Module**
Sesion 1	Information collection	Verbal and visual learning	Verbal and visual learning
Sesion 2	Interpretation and discussion	Memory	Memory
Sesion 3	Interpretation and discussion	Social perception (ToM)	Memory
Sesion 4	Title finding	Social perception (ToM)	Verbal and visual learning
**Third Block**	**Module 3**	**Module C**	**Module C**
Sesion 1	Repetition/paraphrasing	Thinking / problem solving	Thinking / problem solving
Sesion 2	Questions and answers	Problem-solving	Thinking / problem solving
Sesion 3	Asking questions	Problem-solving	Thinking / problem solving
Sesion 4	Focussed communication	Problem-solving	Thinking / problem solving
**Fourth Block**	**Module 4**	**Module C / Module D**	**Module C /Module D**
Sesion 1	Cognitive processing	Social schemata	Thinking / problem solving
Sesion 2	Cognitive Processing	Social schemata	Thinking / problem solving
Sesion 3	Role-playing games	Working memory	Working memory
Sesion 4	Role-playing games	Working memory	Working memory
**Fifth Block**	**Module 5**	**Module D**	**Module D**
Sesion 1	Problem identification	Attribution	Working memory
Sesion 2	Generating solutions	Attribution	Working memory
Sesion 3	Generating solutions	Attribution	Working memory
Sesion 4	Implementing solutions	Emotion regulation	Working memory

### Randomization and Switch Procedure

Following baseline assessment, patients will be randomly assigned (1:1:1 fashion) to either IPT or INT (active interventions) or Cogpack (control intervention). We expect six to eight participants in each treatment arm. Through randomization, there is the possibility that some participants will experience difficulties with their assigned treatment group. In such cases, patients will be permitted to switch to another treatment arm after 2 weeks. The sole criterion for switching intervention arm is patient preference due to excessive, respectively insufficient demands. We have chosen not to formulate explicit criteria for switching intervention arm for two reasons; first of all to empower patients in their commitment to therapy; second to prevent bias through the delayed selection of participants.

**Figure 1:**
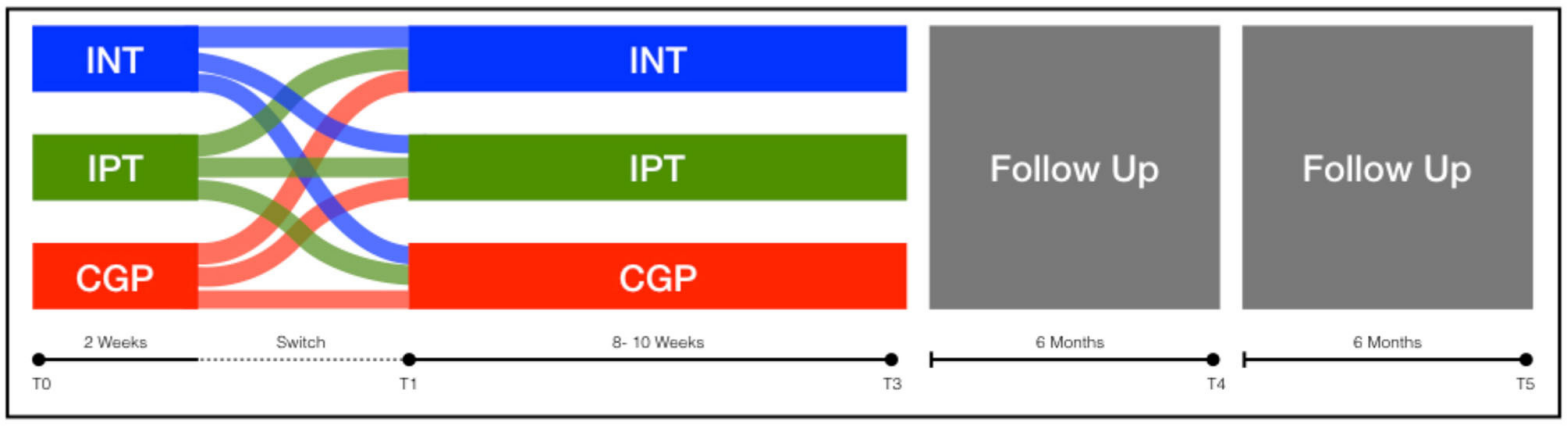
Study flow chart.

After participation in a minimum of 16 sessions (60–90 min each) in one treatment arm, the intervention will be completed; follow-up assessments will be carried out at 6 and 12 months. For a Study- Flow diagram (see Table 3 and Figure 1).

**Table 3:** Schedule of enrolment, interventions, and assessments.

	**Study period**						
	**Enrolment**	**Allocation**	**Post allocation**				**Close-out**
**Time Point**	**–t_**1**_**	**t_**0**_**	**t_**1**_**	**t_**2**_**	**t_**3**_**	**t_**4**_**	**t_**5**_**
**Enrolment**							
Eligibility screen	X						
Informed consent	X						
Allocation		X					
Switch				X			
**Interventions**							
INT			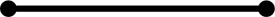				
IPT			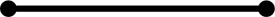				
Cogpack			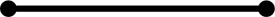				
**Assessments**							
Demographic data	X	X					
Psychopathology			X	X	X	X	X
Psychosocial functionality			X	X	X	X	X
Neurocognition			X	X	X	X	X
Medication			X	X	X	X	X
Safety				X	X		

### Ethical and Regulatory Considerations

The study will be conducted in Switzerland in accordance with current regulations. The ethics committee of the Canton of Zurich approved the study protocol (BASEC Nr.: 2017- 01351). The study protocol was registered in clinicalTrials.gov (NCT03316664; 17.10.2017). Written informed consent will be obtained from the participants before study enrollment.

### Assessments of Outcomes, Raters

Study measurement and outcomes will be assessed by raters blinded regarding group allocation and treatment of the participants. Raters have a psychology degree and training in research methods. All raters have been systematically trained in the use of the study instruments. To ensure raters are blind to treatment arm, they will have no access to patient or study records beyond the strict requirements for rating.

Furthermore, rating sessions will be conducted outside the ward and treatment facilities. In the case of a participant revealing treatment arm allocation to the rater this will be documented. Following the completion of the study, such violations of blinding will be analyzed to determine whether they modified the results.

### Assessment of Outcome, Instruments

Primary outcomes are change in symptom load as measured by the Positive and Negative Syndrome Scale (PANSS) (39), in level of functioning as assessed by the MINI ICF- APP (40) and performance in the Matrics Cognitive Consensus Battery (MCCB) (32). Other outcomes of interest are symptoms and complaints as measured by the Brief Symptom Inventory (BSI) (41), overall severity (or improvement) as measured by the Clinical Global Impression (CGI) (42) scales, overall functionality as measured by the Global Assessment of Functioning (GAF) (43), along with overall mental health as measured by the Health of the Nation Outcome Scales (HoNOS) (44). An overview of the outcome measurements is given in Table 4. Besides these measurement instruments, basic demographic, and clinical characteristics together with medication will also be collected.

**Table 4:** Outcome measurements.

**Test instrument**	**Description**
Positive and Negative Syndrome Scale (PANSS)	The PANSS is a semi-structured interview designed to measure the severity of psychopathology in adult patients with a psychotic disorder, mainly schizophrenia and schizoaffective disorder (39, 45). It measures symptoms in three domains, positive (seven Items), negative (seven items), and general-non-specific symptoms (16 items). Each item is rated on a seven-point Likert scale: from 1 (not present), and from 2 (present) to 7 (very severe). The PANSS range is from 30 to 210 (39).
Mini ICF—APP	The Mini-ICF-APP was developed (40), as a short observer-rated scale for assessing the level of functioning according to the International Classification of Functioning, Disability, and Health (ICF). It assesses 13 domains of functioning, with anchor definitions provided in the manual. Each item is rated on a five-point Likert scale from 0 (no disability) to 4 (total disability).
MATRICS Consensus Cognitive Battery (MCCB)	The MATRICS (Measurement and Treatment Research to Improve Cognition in Schizophrenia) Consensus Cognitive Battery was developed to provide a relatively brief evaluation of key cognitive domains relevant to schizophrenia and related disorders (46). The test Battery includes 10 tests which represent seven different cognitive domains; the test battery is administered as a unit in a standard order. To facilitate a valid interpretation of test scores psychometric properties and normative data were also analyzed (47).
Clinical Global Impression (CGI)	The CGI scale is a brief, easy to use and pragmatic tool for the assessment of psychiatric illness severity and changes over time (42, 48, 49). The CGI consists of three subscales: 1. Severity of Illness (CGI-S), 2. Global Improvement (CGI-I), and 3. Efficacy Index (CGI-E). CGI-S and CGI-I have a seven-point Likert scale response format. It ranges from 1 representing the “healthy subject” to 7 the “extremely ill subject.” The CGI-I ranges from 1 “significant improvement” to 7 “most severe deterioration,” whereby a score of 4 indicates no change. The CGI-E relies on several parameters from whom an index is calculated.
Global Assessment of Functioning (GAF)	The GAF is an observer-rated, 100-point single item scale that rates overall functioning on a continuum from mental health to mental illness (43). The scale ranges from 1 (representing the most impaired individual) to 100 (representing the healthiest individual), divided into (10) deciles, 0 denoting insufficient information to make a judgment.
Health of the Nation Outcome Scales (HoNOS)	The HoNOS is an observer-rated scale and consists of 12 items with a five-point Likert scale response format from 0 (no problems) to 4 (severe/very severe problems). Scores above two are considered clinically significant. The items were combined into four dimensions; each subject can be evaluated on items, subscale scores and the total score (44, 50).

### Statistical Analysis

We calculated our required sample size using G^*^Power 3.1 (51) (ANOVA: Repeated measures, three groups, four measurement points; effect size F = 0.25; < = 0.05; Power = 0.80; number of groups = 3). Based on that calculation, at least 90 participants (30 in each study arm) completing the intervention are required in order to detect small to moderate size differences. Cohen's d (effect size) will be used to compare the percentage of variation in the groups before and after treatment.

All data sets of participants will be analyzed, on an intention to treat basis. Data at baseline, second week and after the intervention, as well as at 6 and 12-months post-intervention will be considered (see Table 3). The demographic and clinical characteristics of the sample will be compared at baseline using an analysis of variance (ANOVA), except for gender and age, which will be assessed using an analysis of covariance (ANCOVA). To compare the overall effect of treatment over time in the three groups, data from the full intent to treat sample will be analyzed using a repeated-measures analysis of variance (ANOVA) with treatment as the intergroup factor and time as the intrasubject factor. *Post-hoc* analyses will be performed using Student's *t*-test for intergroup comparisons. Reported adverse effects and safety-related events will be analyzed separately as will withdrawal from the study or treatment group changes.

### Participants Switching Groups, Drop-Outs, and Missing Data

Drop-outs will be replaced until the calculated number of participants has completed the intervention; all enrolled participants will be allowed to complete the intervention. Patient switching therapeutic arm will be considered as drop-outs and will be replaced correspondingly; however, they will be allowed to complete the intervention and outcomes will be assessed as scheduled. For all dropouts, an additional intention to treat, and last observation carried forward analysis will be performed.

In contrast to drop-outs the assessment of outcomes and observation of participants switching groups will be continued. Data prior to switching will be analyzed and reported separately in order to avoid bias duplication of results for a particular participant.

The outcomes of those participants who completed the intervention after switching groups will undergo a *post-hoc* analysis with those who completed their treatment intervention without switching. A multivariate analysis of variance (MANOVA) will be performed, taking into account the switch of intervention together with outcomes measured pre- and post-intervention; i.e., ignoring the outcomes at week two for those not switching intervention arm. Groups for analysis will be constructed according to the number of participants actually switching interventions: the effect of the four first sessions will be categorized according to the change and included in the analysis.

If a participant withdraws from the study, his data will be anonymized, and his name will be deleted permanently from the study.

### Data Sharing and Publication

After completion of the study, a report for publication in a peer-reviewed journal will be prepared. The manuscript will be edited/compiled according to the CONSORT statement recommendation (52–54). The study protocol has been registered at: www.clinicaltrials.gov (ID: NCT0331664; 17.10.2017). The study protocol was written according to the SPIRIT 2013, statement for reporting of trial protocols (55) and the TIDieR guidelines (56).

### Expected Outcomes

We expect all treatment arms to show similar overall rates of improvement; with differences in specific domains. Regarding symptom load, we do not expect any treatment arm to perform significantly better than the others. Regarding psychosocial functioning, we expect INT and IPT to be superior to CGP. Regarding cognitive abilities, we expect that patients participating in INT will perform better than both CGP and IPT participants.

Regarding the feasibility and implementation of the treatment arms, we expect similar retention rates in all treatment arms. Moreover, we anticipate that chronic and lower functioning participants will switch to CGP; whereas higher functioning participants may switch to INT, with lower fluctuation in IPT. Consequently, we anticipate slightly higher retention rates for IPT, followed by INT and CGP. For an overview of the expected outcomes (see Table 5).

**Table 5:** Expected outcomes.

	**Intervention**		
**Expected outcomes**	**INT**	**IPT**	**Cogpack**
Overall improvement (CGI)	+	+	+
Overall functionality (GAF)	+++	++	+
Prescribed medication	+	++	+++
Drop out-switch of treatment group	+++	++	+
Completion of therapy	++	++	+++
Symptom load (PANSS)	+	+	+
Symptom perception (BSI)	++	++	+
Psychosocial functioning (mini ICF)	++	++	+
Psychosocial functioning (HoNOS)	++	++	+
Neurocognitive performance (MCCB)	+++	+	++
Speed of processing	++	+	+++
Verbal learning	+++	++	+
Working memory (non- verbal)	++	+	++
Working memory (verbal)	++	++	+
Reasoning and problem solving	+++	++	++
Visual learning	++	+	+++
Social cognition	++	++	+
Attention/Vigilance	+++	++	+++

## Discussion

Schizophrenia is a severe chronic disorder; those affected are frequently not able to cope with everyday challenges. Despite advances in treatment and an increase in treatment options the proportion of patients with schizophrenia who fully recover remains practically unchanged, this holds especially true for those requiring hospitalization for treatment (4, 57), however, for methodological reasons, those severely affected by a psychiatric disorder (i.e., those requiring hospitalization) are frequently excluded from studies testing new treatment interventions (58, 59).

The implementation of a new treatment intervention in clinical practice and moreover in an inpatient setting is a major challenge presenting a number of methodological and logistic problems. Firstly, patients hospitalized for treatment are generally more heterogeneous, with several co-morbid conditions, as well as being more severely affected than those typically included in studies. Especially patients with psychotic disorders who are more severely affected tend to have less insight and treatment motivation; consequently, they are less likely to enroll in therapy. Furthermore, the very negative and cognitive symptoms which require treatment may themselves interfere with treatment. Participants may, therefore, be unable to cope with the demands of therapy, leading to demotivation and frustration, which may lead to drop-out from therapy. We have therefore chosen to compare three treatment interventions with similar therapeutic targets and approaches, but differing participation (for patients) and implementation (for therapists) thresholds.

In clinical practice, factors relating to a patient which may affect the therapeutic outcome are considered before initiating treatment. This procedure is, however, incompatible with a randomized assignment of treatment. Furthermore, it makes it impossible to determine if a treatment is really feasible for a group of participants. Therefore, we chose to allow a switch of treatment groups after four sessions, a challenging methodological alternative which should, however, facilitate participants finding the appropriate therapy whilst allowing us to achieve our study goals. The decision to switch from one intervention to another is entirely the participant's choice. This approach strengthens the commitment of participants toward the therapy (60), by reducing feelings of failure or inadequacy which may be raised by psychometric and neurocognitive testing (61). Furthermore, we consider this approach will reduce a potential delayed selection bias.

Taking into account that participants switching treatment arms will continue their treatment, we have decided to continue the assessment of outcomes. Baseline severity will, in such cases, correspond to the time when the main therapy began. This has as a consequence that in addition to the three original treatment arms other treatment arms emerge, namely those of switching from one treatment to another. Since we are not able to accurately foresee how many participants will use this option, and if so to which treatment option they will switch, we have decided to include this in a *post-hoc* analysis. To avoid bias and duplication, we will consider participants switching groups as drop-outs in the primary analysis.

The assessment of outcomes uses a variety of instruments measuring symptom load, neuro-cognitive performance and psychosocial functionality since we expect each treatment arm to show a unique response profile (11). For symptom load, we have chosen to use the PANSS, since it is considered the most specific and validated scale for measuring change in patients with a schizophrenic psychosis (62, 63). The MCCB is regarded as the standard test battery for use with patients diagnosed with schizophrenia; it is claimed as both robust to learning effects and sensitive to change (47). Taking into account that improvements in symptomatology and neuro-cognition should lead to increased autonomy, we have chosen the mini-ICF-APP to assess psychosocial-functioning independently of the main diagnosis (40, 64, 65). Using this set of outcome measures, we expect to assess all facets of the interventions, whilst facilitating the interpretation and comparison of our results with those from previous studies (63).

We anticipate an overall improvement in all participants, regardless of group allocation. This may be attributable to pharmacological treatments administered to the participants. For the same reason, we do not anticipate significant differences in symptom load. However, we do not exclude to find differences in dose equivalents of administered medication, primarily antipsychotics and benzodiazepines. Furthermore, we hope for a reduction of polypharmacy. In order to quantify this effect; medication, respectively dose and dose equivalents, will be taken into account as possible confounders (66). Pharmacological treatment will not be restricted since we consider withholding or delaying pharmacological treatment for methodological or design purposes to be unacceptable; furthermore, in clinical practice, psychological interventions are used as an add-on to pharmacological therapy (11). Offering just a single psychotherapeutic treatment could be ineffective for some patients and at worst detrimental, which could lead to treatment abandonment (67). An intervention which is neither accepted nor tolerated by participants should not be imposed on them.

The allocation of a patient to a treatment arm which does not meet his needs is considered to be unacceptable and may have detrimental effects. To avoid this situation, we have decided to allow participants to switch groups under particular conditions. We consider this approach to be both appropriate and compatible with the trial objectives since we are also interested in evaluating feasibility. In order to minimize the effect of group changes on the primary and secondary outcomes of the study, and to have similar treatment duration and number of sessions, we have chosen to conduct a second assessment prior to a (potential) change of groups. We consider 2 weeks, respectively four sessions, sufficient to determine the suitability and safety of a therapy. INT in outpatient setting shows low drop-out rates and high rates of attendance which indicates a high level of acceptance and motivation and lends support feasibility (29). Another aim of this study is to evaluate whether this also applies to inpatient settings.

Another challenge of this trial was the adaptation of a treatment (INT) designed for outpatients to an inpatient setting (29, 38). The hierarchical structure of the intervention, the density of the therapeutic sessions, and the duration of the therapy had to be taken into account together with the fact that the original therapeutic design foresees closed therapeutic groups (27). For its implementation in an inpatient setting, we subdivided the intervention into modules or blocks comprising of four sessions, allowing the participants to join the therapy at specified times thus allowing for (semi)closed groups. The disadvantage of this approach lies in the hierarchical structure of both INT and IPT interventions (23, 27), which leads participants potentially starting with a more challenging topic; to minimize this effect blocks or modules also had a bottom-up structure allowing new participants to integrate quickly into the group.

Lower functioning patients with low functioning, including those with chronic schizophrenia, seem to benefit from higher frequency psychotherapy (21, 68). We, therefore, concluded that two weekly sessions of 90 min would fulfill this demand. Taking into account the usual length of stay in our unit (6 to 8 weeks), together with current guidelines (17–19) we have chosen to offer at least 16 sessions to participants. For methodological reasons it was not possible to condense both therapeutic interventions to this number of sessions, therefore IPT, INT and CGP have been adapted to 20 sessions programs, respecting the specifications of the treatment manuals.

We considered this to be compatible with the average length of stay in our unit, since some patients remain in treatment for more than 8 weeks. Participants are also encouraged to continue participation after discharge. Although we consider that the completion of a treatment programme to be an important factor determining response, evidence suggests that response is not associated with treatment duration and number of sessions (10).

There is reason to believe that the three treatment arms will differ from one another due to top-down and integration effects on multiple domains. In respect of social- and neuro-cognition, we expect INT and IPT to outperform CGP (36, 69). Nevertheless, we cannot rule out that CGP may also lead to improvements in neuro-cognitive abilities, in particular MCCB domains tested on the computer due to learning and practice effects (12, 70). We expect INT participants to achieve a higher psychosocial performance than CGP, but similar to those of IPT. Although INT and IPT have common roots, applying similar technics for social cognition (23, 27, 37), we expect INT to deliver better outcomes due to its higher complexity and integrative approach (see Table 5).

In our study, we wish to evaluate the feasibility of a psychotherapeutic intervention in a unit specialized for the treatment of chronic and low functioning patients with a schizophrenic psychosis. Therefore, the effort required to implement such regular group psychotherapy will also be taken into account. The IPT treatment programme is manualized and has traditionally been delivered by nursing staff after an introductory seminar and workshop (23). The implementation of INT is more challenging since it also requires a trained psychotherapist (27). CGP, on the other hand, is easily implemented and requires practically no training to deliver (26, 71, 72). However, professional experience is a significant factor predicting therapy outcome. Moreover, the therapist variable is an essential factor relating to motivation to participate in the therapy and influencing drop-out rates (73–75). Consequently, all therapists running groups in this study will receive regular supervision and training.

## Author Contributions

MR wrote the manuscript. DM contributed to the study design and manuscript. ES, SV, and SE contributed to the study design, study protocol and manuscript. All authors contributed to the article and approved the submitted version.

## Conflict of Interest

The authors declare that the research was conducted in the absence of any commercial or financial relationships that could be construed as a potential conflict of interest.
